# Ketogenic effects of medium chain triglycerides containing formula and its correlation to breath acetone in healthy volunteers: a randomized, double-blinded, placebo-controlled, single dose-response study

**DOI:** 10.3389/fnut.2023.1224740

**Published:** 2023-09-27

**Authors:** Kentaro Nakamura, Keisuke Hagihara, Naoko Nagai, Ryuichiro Egashira, Mariko Takeuchi, Mai Nakano, Hitomi Saito, Misaki Moriguchi, Satoko Tonari, Hisako Fujii, Akimitsu Miyake, Yusuke Omae, Kinya Ashida

**Affiliations:** ^1^Co-Creation Center, Meiji Holdings Co., Ltd., Tokyo, Japan; ^2^Department of Advanced Hybrid Medicine, Osaka University Graduate School of Medicine, Osaka, Japan; ^3^Division of Nutritional Management, Osaka University Hospital, Osaka, Japan; ^4^Department of Drug and Food Evaluation, Osaka City University Graduate School of Medicine, Osaka, Japan; ^5^Department of Medical Innovation, Osaka University Hospital, Osaka, Japan

**Keywords:** medium chain triglycerides, ketogenic diet, single dose study, dose response, breath acetone

## Abstract

The efficacy of low-carbohydrate, high-fat diets, such as ketogenic diets, for cancer patients is of research interest. We previously demonstrated the efficacy of the ketogenic diet in a case study in which medium-chain triglycerides (MCTs) or MCT-containing formula (ketogenic formula) was used as a supplement to increase blood ketone bodies. However, little is known about the amounts needed to induce ketogenic effects and about the usefulness of monitoring of breath acetone. To investigate the pharmacokinetics of MCTs and their metabolites, blood ketone bodies and breath acetone, 24 healthy subjects received one of four single oral doses of the ketogenic formula (equivalent to 0, 10, 20, and 30 g of MCTs) under fasting conditions. Total blood ketone bodies, β-hydroxybutyrate, octanoic acid, and decanoic acid were increased in a dose-dependent manner. The ketogenic effect was considered to depend on octanoic and decanoic acids, because a positive correlation was observed between them. A strong positive correlation was also observed between total serum ketone bodies and breath acetone at each time points. Therefore, monitoring breath acetone levels seems a less invasive method to predict blood concentrations of ketone bodies during ketogenic diet therapy.

**Clinical trial registration:**https://rctportal.niph.go.jp/en/detail?trial_id=UMIN000032634, UMIN-CTR UMIN000032634.

## 1. Introduction

The ketogenic diet is a high-fat, low-carbohydrate diet with adequate amounts of protein that is designed to increase blood ketone bodies (1). The ketogenic diet is a nutrition therapy for the treatment of epilepsy (2). Recently, this diet has been reported to exert various effects on our health, such as losing weight, improving lipid profiles and enhancing cognitive functions (3–6). In addition, the ketogenic diet and ketone bodies have been shown to have a therapeutic potential in several pathological conditions, such as diabetes, polycystic ovary syndrome, acne, neurological diseases, respiratory and cardiovascular diseases, and cancer (7–9). Although the ketogenic diet is inexpensive, fairly easy to implement, it is not well tolerated in the long term (10). Dietary modifications improve the tolerability of the ketogenic diet. Besides carbohydrate restriction, ingesting medium-chain triglycerides (MCTs) is a common method to increase blood ketone bodies (11). MCTs in commercial products are mainly composed of medium-chain fatty acids, octanoic acid (C8) and decanoic acid (C10), and are rapidly digested and absorbed after ingestion and are converted to ketone bodies in the liver (12). A ketogenic diet using MCTs could allow a higher carbohydrate content in meals and achieve sufficient ketone body production (10).

We previously reported a case study suggesting that the ketogenic diet may be a promising supportive therapy for patients with various types of advanced cancer (13). In this ketogenic diet, the ketogenic formula, which contains MCTs, was used as supplements to enhance blood ketone bodies. The ketogenic formula is a special infant formula with a high-fat, low-carbohydrate composition, which has long been used for ketogenic dietary therapy for infants with congenital metabolic disorders and refractory epilepsy in Japan (2, 14). However, it has not been clear how much formula is needed to increase blood ketone body levels in adults. In our ketogenic diet regimen, the amount of ketogenic formula used was clinically determined based on practical experience, while referring to blood levels of ketone bodies (13). In this study, to establish an effective ketogenic diet regimen for adult patients, we investigated the pharmacokinetics of blood ketone bodies in healthy individuals supplemented with a single administration of ketogenic formula. In addition, we investigated the correlation between blood ketone bodies and breath acetone to evaluate the monitoring breath acetone.

## 2. Materials and methods

### 2.1. Ethics statement

This study was conducted with the approval of the ethical review committees at each facility of Osaka University, Osaka City University, medical institution for clinical trial implementation, and Meiji Co., Ltd., which is a joint research institute (approval numbers 17443, 4060, and 2017-022, respectively), and was registered in the University Hospital Information Network (UMIN) clinical trial system prior to enrollment of subjects (UMIN000032634).

### 2.2. Study participants

The inclusion criteria were as follows: the subject signed a written informed consent form to participate in the study, was a male aged between 20 and 40, and had a body mass index between 18.5 and 25. The exclusion criteria were as follows: the subject was a smoker; was receiving drug treatment; had digestive abnormalities; was allergic to milk, soybeans, or pork; had lactose intolerance; was currently on a low-carbohydrate diet or a ketogenic diet; or had an abnormal value in blood biochemical tests (fasting blood glucose, hemoglobin A1c, aspartate aminotransferase, alanine aminotransferase, alkaline phosphatase, γ-glutamyl transferase, blood urea nitrogen, uric acid, creatinine, triglycerides, high-density lipoprotein cholesterol, C-reactive protein, red blood cells, white blood cells, hemoglobin, hematocrit, and platelet count).

### 2.3. Test formula

The subjects were provided the ketogenic formula (Ketonformula^®^ 817-B, Meiji Co., Ltd., Tokyo, Japan) and the placebo formula. The ketogenic formula is a special infant formula with a high-fat, low-carbohydrate content, which is formulated with MCTs with a ketogenic ratio of 3:1. In a placebo formula, the MCTs contained in the ketogenic formula were replaced with long-chain triglycerides (LCTs). Seventy-five grams of powder was dissolved in water on the test day, and the total volume was prepared to 300 mL. The compositions of the formulas are listed in Table 1.

**Table 1:** Nutritional contents of the test formulas (per 75 g).

Ingredient		Ketogenic formula	Placebo formula
Carbohydrates (g)		6.7	
Proteins (g)		11.3	
Lipids (g)	MCTs (g)	30.0	0.0
	LCTs (g)	24.3	54.3
Ash (g)		2.7	
Others		Vitamin mix, mineral mix	
Ketogenic ratio		3.0	
Carbohydrates (% kcal)		5	
Proteins (% kcal)		8	
Lipids (% kcal)		87	

MCTs, medium-chain triglycerides; ketogenic ratio, the ratio of the weight of lipids to the weight of carbohydrates and proteins.

### 2.4. Study design

This study was conducted in a double-blind, randomized, parallel-group comparative manner. After consent was obtained, the subjects were randomly allocated using the block method. Twenty-four subjects were randomly assigned to four groups: (1) placebo (placebo formula 75 g; the amount of MCTs was 0 g); (2) low-dose (ketogenic formula 25 g + placebo formula 50 g; the amount of MCTs was 10 g); (3) medium-dose (ketogenic formula 50 g + placebo formula 25 g; the amount of MCTs was 20 g); and (4) high-dose (ketogenic formula 75 g; the amount of MCTs was 30 g).

The subjects visited the hospital without breakfast on the test day. Medical interviews and blood sampling were performed, and breath acetone concentration, vital signs, and the Gastrointestinal Symptom Rating Scale (GSRS) were measured as baseline data. Next, the test formula (about 300 mL) was ingested once. Blood was collected, and breath acetone concentration was measured at 0.5, 1, 1.5, 2, 3, 4, and 6 h after ingestion of the test formula. In addition, a medical interview was conducted at 3 and 6 h, and the GSRS score and vital signs were measured at 6 h. The GSRS score is a validated questionnaire for measurement of the quality of life of gastrointestinal symptoms regarding reflux, abdominal pain, indigestion, diarrhea, and constipation. The subjects were restricted from taking a meal in addition to the formula until the study was completed.

### 2.5. Measurements

The blood samples were sent to LSI Medience Corporation^1^ for measurements of the concentrations of β-hydroxybutyrate (BHB), acetoacetic acid (AcAc), and glucose. The term “serum total ketone bodies (serum TKB)” represents the sum of BHB and AcAc concentrations in serum. We also measured the serum concentrations of medium-chain fatty acids: octanoic (C8), decanoic (C10), and dodecanoic (C12) acid. Serum samples were methyl esterificated using a Fatty Acid Methylation Kit (Nacalai Tesque, Kyoto, Japan) according to the manufacturer’s protocol. Methylated medium-chain fatty acids were quantified using gas chromatography–mass spectrometry (GC–MS) (SHIMADZU, Kyoto, Japan). We measured breath acetone concentration using a breath acetone-measuring device (NTT Docomo, Japan).

### 2.6. Sample size calculation and statistical analysis

The sample size was calculated based on our previous study, in which changes in plasma ketone bodies were tracked after a single administration of 50 g of the ketogenic formula to healthy aged subjects without cognitive impairment (5). We calculated that enrollment of six subjects in each group was required with an α of 5% and a power of 80%.

The data presented in the tables and figures are expressed as means ± standard deviation. Statistical analysis software R3.4.4 was used. The dose proportionality of maximum concentration (Cmax) and the area under the curve (AUC) were evaluated using a power model. The point estimate of the slope of the regression line and the confidence interval (CI) were calculated, and the confidence coefficient was 95%. Dose proportionality was concluded if the 95% CI included 1. Correlations between total serum ketone bodies and medium-chain fatty acids and breath acetone were investigated by Pearson’s correlation coefficient test. Differences were considered significant at *p* < 0.05.

## 3. Results

### 3.1. Subjects

Twenty-eight subjects were recruited, and four did not meet the inclusion criteria; therefore, 24 subjects were enrolled in the study (Supplementary Figure S1). The subjects were randomly divided into the placebo group (*n* = 6), low-dose group (*n* = 6), medium-dose group (*n* = 6), and high-dose group (*n* = 6). One subject assigned to the medium-dose group did not participate on the test day, so that the medium-dose group was investigated at *n* = 5. The demographic characteristics of the subjects at baseline are summarized in Table 2. There were no differences between the groups in height, weight, body mass index, muscle mass, or hematological values.

**Table 2:** Demographic characteristics of subjects at baseline.

Characteristic	Placebo (*N* = 6)	Low-dose (*N* = 6)	Medium-dose (*N* = 5)	High-dose (*N* = 6)
Age (y)	23.33 ± 5.28	23.83 ± 7.47	28.00 ± 8.34	20.67 ± 0.82
Height (cm)	172.83 ± 4.09	171.95 ± 3.37	175.96 ± 2.54	169.23 ± 7.60
WBC (counts/μL)	5450.00 ± 1125.61	5750.00 ± 1262.93	4980.00 ± 1251.80	5566.67 ± 1048.17
RBC (×10^4^/μL)	514.50 ± 15.60	506.33 ± 20.39	497.40 ± 28.88	537.67 ± 10.09
Hb (g/dL)	15.33 ± 0.92	15.65 ± 0.52	15.18 ± 1.03	16.30 ± 0.70
Ht (%)	46.32 ± 1.80	47.27 ± 1.19	45.74 ± 2.17	49.05 ± 1.16
PLT (×10^4^/μL)	25.45 ± 2.21	24.28 ± 3.60	23.98 ± 4.05	24.02 ± 5.59
AST (U/L)	16.17 ± 1.47	18.17 ± 4.40	16.80 ± 2.39	20.00 ± 2.76
ALT (U/L)	14.67 ± 4.80	16.17 ± 8.68	12.00 ± 4.74	17.00 ± 7.97
γ-GTP (U/L)	22.50 ± 9.09	23.00 ± 13.78	20.80 ± 10.85	20.50 ± 4.72
ALP (U/L)	192.83 ± 35.47	187.83 ± 34.95	171.80 ± 40.85	203.83 ± 40.07
BUN (mg/dL)	11.90 ± 2.34	13.67 ± 3.13	13.68 ± 2.91	10.82 ± 2.09
CRE (mg/dL)	0.82 ± 0.12	0.80 ± 0.09	0.91 ± 0.08	0.86 ± 0.12
UA (mg/dL)	6.47 ± 0.56	6.33 ± 1.07	5.72 ± 0.90	6.05 ± 0.95
TG (mg/dL)	58.00 ± 11.87	73.33 ± 32.42	62.20 ± 28.55	88.67 ± 13.00
HDL-C (mg/dL)	54.83 ± 13.38	56.83 ± 5.49	55.80 ± 15.48	51.50 ± 8.07
Glucose (mg/dL)	81.33 ± 5.96	82.17 ± 6.85	81.60 ± 4.77	79.33 ± 8.82
HbA1c (%)	5.25 ± 0.19	5.27 ± 0.16	5.24 ± 0.27	5.22 ± 0.17
CRP (mg/dL)	0.04 ± 0.05	0.02 ± 0.01	0.02 ± 0.01	0.02 ± 0.02

Values are presented as means ± standard deviation. WBC, white blood cells; RBC, red blood cells; Hb, hemoglobin; Ht, hematocrit; PLT, platelet count; AST, aspartate aminotransferase; ALT, alanine aminotransferase; γ-GTP, γ-glutamyl transferase; ALP, alkaline phosphatase; BUN, blood urea nitrogen; CRE, creatinine; UA, uric acid; TG, triglycerides; HDL-C, high-density lipoprotein cholesterol; HbA1c, hemoglobin A1c; CRP, C-reactive protein.

### 3.2. Blood ketone bodies

**Figure 1:**
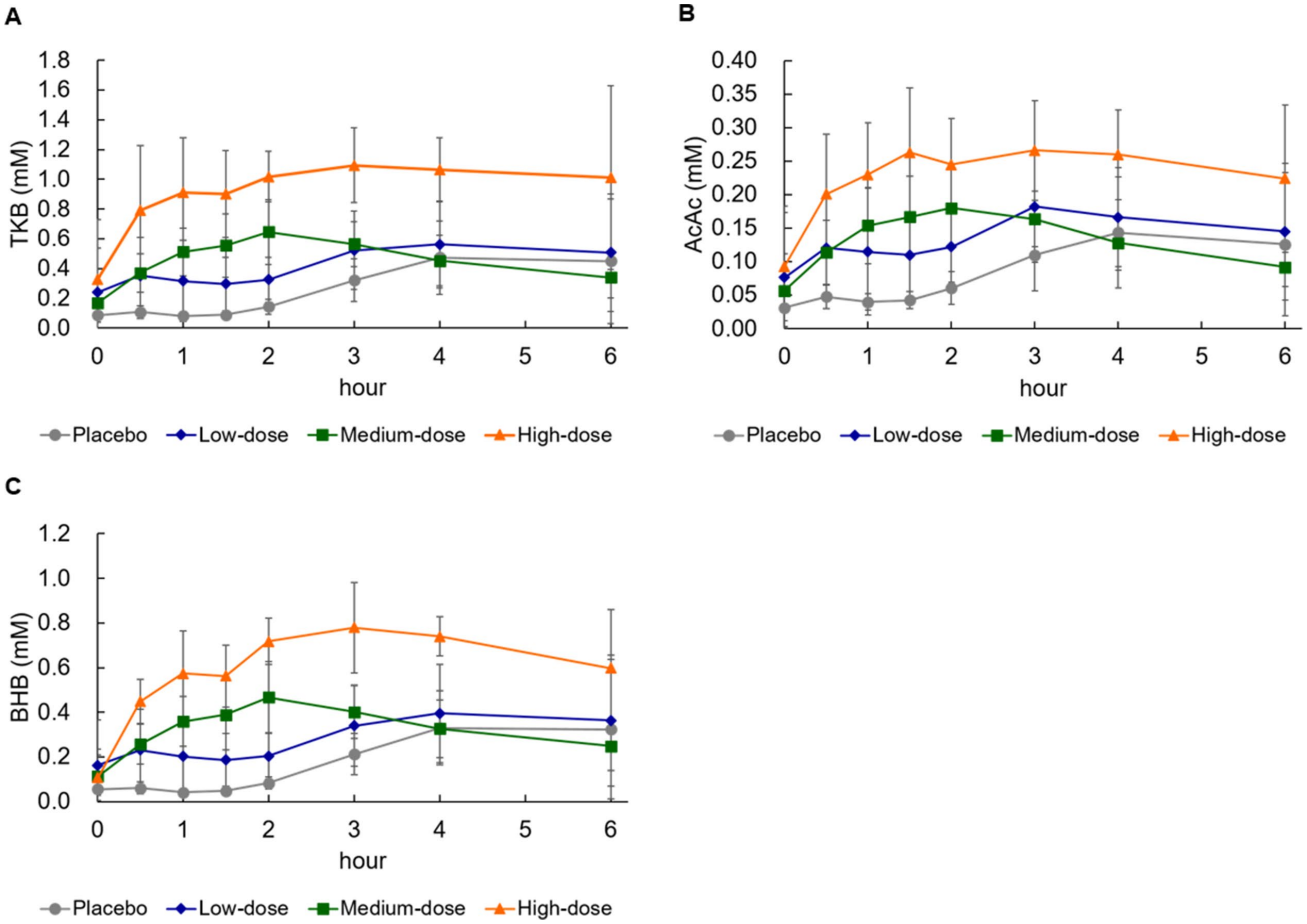
Changes in blood concentration of ketone bodies and glucose. **(A)** Total ketone bodies (TKB), **(B)** acetoacetic acid (AcAc), **(C)** β-hydroxybutyrate (BHB). Values are means ± standard deviation.

As shown in Figure 1A, in the high-dose group, serum TKB began to increase from 30 min after ingestion, and reached the peak concentration at 3–4 h (Table 3; Tmax: 3.7 ± 1.4 h). In addition, an increase in serum TKB was observed in the medium-dose group from 30 min after ingestion, which reached the peak concentration at 2 h after ingestion. However, the increase in serum TKB was delayed and reached the peak concentration only at 4–6 h in the low-dose and placebo groups. Next, using a power model, we analyzed whether the ketogenic formula induced serum TKB in a dose-dependent manner. Dose proportionality was demonstrated, as the 95% CI for AUC and Cmax included 1 (Table 3).

**Table 3:** Cmax, AUC, and Tmax of TKB in each group.

TKB	Placebo	Low-dose	Medium-dose	High-dose	Point estimate	95% CI
Cmax (mM)	0.565 ± 0.343	0.635 ± 0.329	0.681 ± 0.198	1.290 ± 0.416	0.64	(0.26, 1.03)
AUC (mM∙h)	1.752 ± 0.959	2.661 ± 1.542	2.832 ± 0.700	5.849 ± 1.764	0.73	(0.33, 1.13)
Tmax (h)	4.7 ± 1.0	4.8 ± 1.3	2.0 ± 0.7	3.7 ± 1.4	-	-

Values are presented as means ± standard deviation. Cmax, maximum concentration; AUC, area under the curve; Tmax, time of Cmax; TKB, total ketone bodies; CI, confidence interval.

An increase in serum AcAc was noted from 30 min after ingestion in the high-dose and medium-dose groups, which reached the peak concentration at 3 h and 1.5–2 h, respectively (Table 4). On the contrary, the serum AcAc reached the peak concentration at 4–6 h in the low-dose and placebo groups. As shown in Table 4, neither the Cmax nor the AUC of AcAc was dose-proportional, because the 95% CI did not include 1 (Table 4).

**Table 4:** Cmax, AUC, and Tmax of AcAc in each group.

AcAc	Placebo	Low-dose	Medium-dose	High-dose	Point estimate	95% CI
Cmax (mM)	0.157 ± 0.089	0.193 ± 0.079	0.190 ± 0.054	0.289 ± 0.098	0.34	(−0.02, 0.70)
AUC (mM∙h)	0.569 ± 0.314	0.860 ± 0.451	0.814 ± 0.188	1.433 ± 0.462	0.46	(0.07, 0.85)
Tmax (h)	4.7 ± 1.0	4.3 ± 1.4	1.8 ± 0.8	3.0 ± 0.7	-	-

Values are presented as means ± standard deviation. Cmax, maximum concentration; AUC, area under the curve; Tmax, time of Cmax; AcAc, acetoacetic acid; CI, confidence interval.

The serum BHB concentration also began to increase from 30 min after ingestion in the high-dose and medium-dose groups, and the BHB reached the peak concentration at approximately 3 h and 2 h, respectively (Table 5). On the contrary, the increase in serum AcAc reached the peak concentration at 4–6 h in the low-dose and placebo groups. When evaluated using a power model, as shown in Table 5, the CI included 1, so it was judged that both Cmax and AUC of BHB were dose-proportional (Table 5).

**Table 5:** Cmax, AUC, and Tmax of BHB in each group.

BHB	Placebo	Low-dose	Medium-dose	High-dose	Point estimate	95% CI
Cmax (mM)	0.409 ± 0.257	0.448 ± 0.248	0.494 ± 0.148	1.014 ± 0.354	0.74	(0.33, 1.14)
AUC (mM∙h)	1.184 ± 0.649	1.800 ± 1.095	2.019 ± 0.522	4.416 ± 1.437	0.83	(0.42, 1.25)
Tmax (h)	4.7 ± 1.0	5.2 ± 1.3	2.0 ± 0.7	3.2 ± 0.8	-	-

Values are presented as means ± standard deviation. Cmax, maximum concentration; AUC, area under the curve; Tmax, time of Cmax; BHB, β-hydroxybutyrate; CI, confidence interval.

### 3.3. Blood medium-chain fatty acids

**Figure 2:**
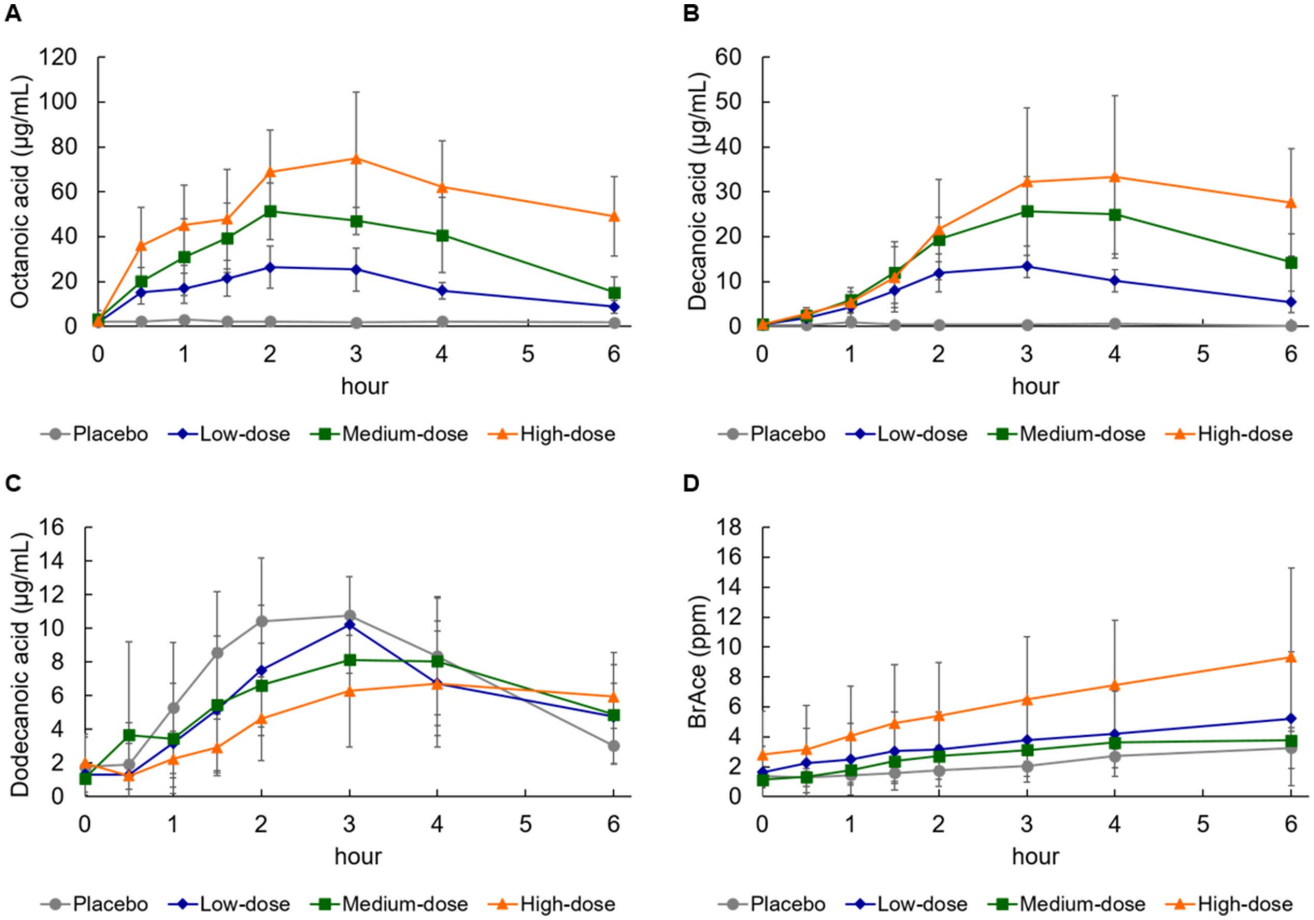
Changes in blood medium-chain fatty acid and breath acetone concentration. **(A)** Octanoic acid, **(B)** decanoic acid, **(C)** dodecanoic acid, and **(D)** breath acetone (BrAce). Values are means ± standard deviation.

Changes in serum octanoic acid concentration were detected 30 min after ingestion in the high-, medium-, and low-dose groups compared with the placebo group (Figure 2A). When evaluated using the power model, Cmax and AUC were judged to be dose-proportional (Table 6). Changes in serum decanoic acid concentration showed similar results, indicating dose proportionality (Figure 2B; Table 6). On the other hand, changes in serum concentrations of dodecanoic acid, which is abundant in the placebo formula, was detected 1 h after ingestion in the placebo group, but no dose proportionality was observed (Figure 2C; Table 6).

**Table 6:** Cmax, AUC, and Tmax of medium-chain fatty acids in each group.

Fatty acid	Placebo	Low-dose	Medium-dose	High-dose	Point estimate	95% CI
Octanoic acid						
Cmax (μg/mL)	3.72 ± 0.61	30.03 ± 8.17	56.04 ± 9.26	79.72 ± 23.84	0.88	(0.63, 1.14)
AUC (μg∙h∙mL^−1^)	13.28 ± 3.68	105.06 ± 23.65	207.91 ± 43.22	333.62 ± 91.46	1.04	(0.80, 1.28)
Tmax (h)	2.1 ± 1.8	2.7 ± 0.5	2.4 ± 1.1	3.0 ± 1.1	-	-
Decanoic acid						
Cmax (μg/mL)	1.23 ± 0.33	14.73 ± 2.97	28.26 ± 8.29	34.90 ± 16.41	0.73	(0.38, 1.09)
AUC (μg∙h∙mL^−1^)	2.95 ± 1.22	50.32 ± 10.07	102.34 ± 26.43	135.72 ± 66.55	0.85	(0.50, 1.19)
Tmax (h)	1.4 ± 0.8	2.7 ± 0.5	3.1 ± 1.0	3.8 ± 0.4	-	-
Dodecanoic acid						
Cmax (μg/mL)	12.98 ± 1.89	10.83 ± 3.50	10.50 ± 3.06	7.28 ± 3.20	–0.35	(− 0.74, 0.03)
AUC (μg∙h∙mL^−1^)	42.40 ± 10.13	35.84 ± 12.49	36.59 ± 14.18	29.43 ± 15.00	–0.21	(− 0.64, 0.22)
Tmax (h)	2.7 ± 0.8	2.8 ± 0.4	2.9 ± 1.4	4.2 ± 1.1	-	-

Values are means ± standard deviation. Cmax, maximum concentration; AUC, area under the curve; Tmax, time of Cmax; CI, confidence interval.

### 3.4. Change in breath acetone concentration

Breath acetone gradually increased and reached the peak concentration at 4–6 h after ingestion of the formula in all groups (Figure 2D; Table 7). Breath acetone had a delayed peak (Figure 2D), unlike serum ketone bodies that reached the peak concentration at 0.5–4 h (Figures 1A–C). When evaluated using a power model, both Cmax and AUC were dose-proportional, although the amount of proportionality was weak (Table 7).

**Table 7:** Cmax, AUC, and Tmax of breath acetone in each group.

Breath acetone	Placebo	Low-dose	Medium-dose	High-dose	Point estimate	95% CI
Cmax (ppm)	3.40 ± 1.31	5.28 ± 4.46	3.82 ± 0.62	9.37 ± 5.93	0.51	(− 0.03, 1.06)
AUC (ppm∙h)	13.15 ± 4.10	21.98 ± 17.33	17.37 ± 3.60	37.88 ± 24.98	0.48	(− 0.05, 1.00)
Tmax (h)	5.3 ± 1.0	5.3 ± 1.8	5.2 ± 1.1	5.7 ± 0.8	-	-

Values are means ± standard deviation. Cmax, maximum concentration; AUC, area under the curve; Tmax, time of Cmax; CI, confidence interval.

### 3.5. Correlations between TKB and medium-chain fatty acids and acetone

**Figure 3:**
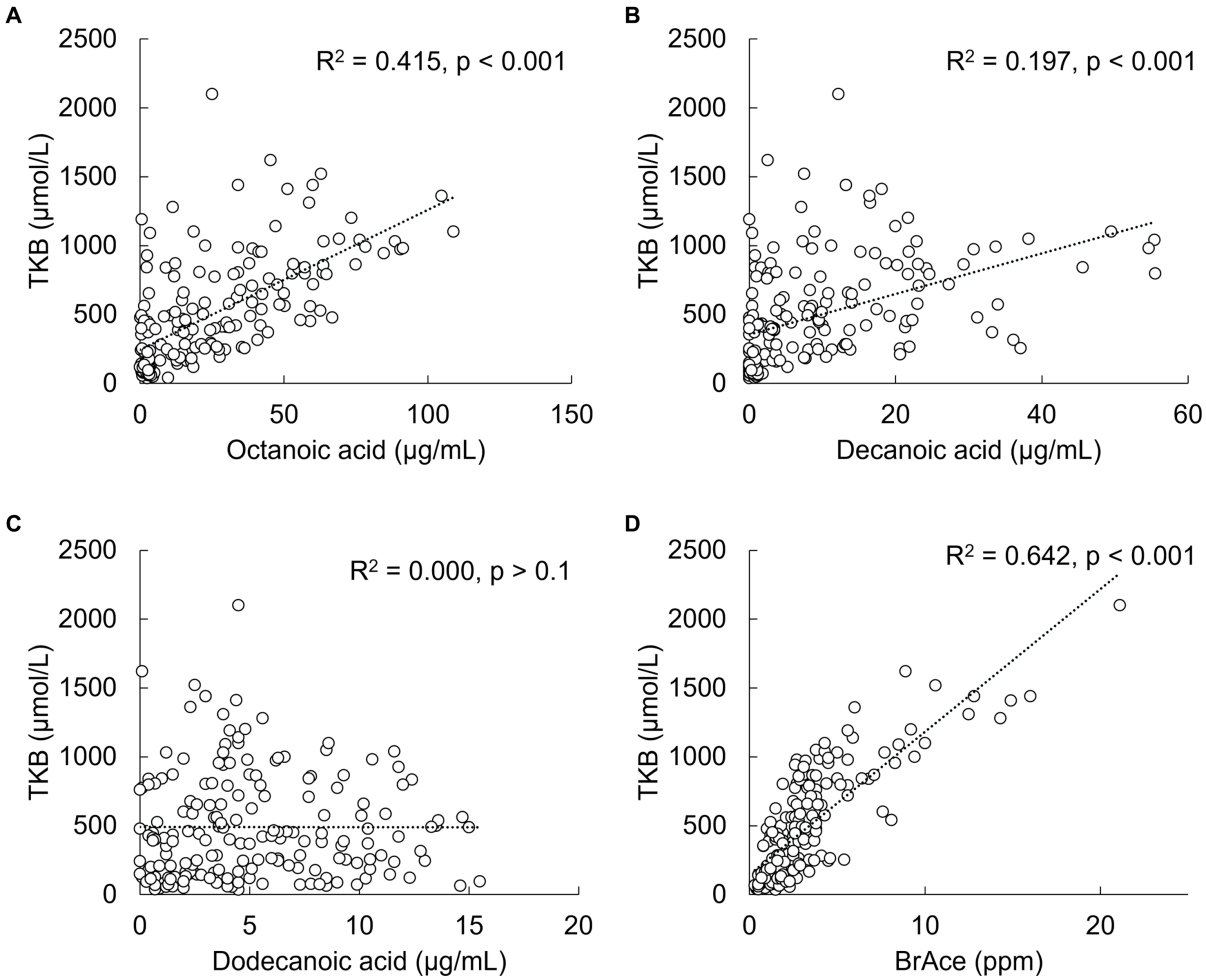
Correlations between total ketone bodies (TKB) and medium-chain fatty acid or breath acetone (BrAce) concentrations. **(A)** Octanoic acid, **(B)** decanoic acid, **(C)** dodecanoic acid, and **(D)** breath acetone.

A statistically significant (*p* < 0.001) positive correlation was observed between serum TKB and octanoic and decanoic acid concentrations, but not with dodecanoic acid concentration (Figures 3A–C). TKB was also significantly correlated with breath acetone, which consists of metabolites of medium-chain fatty acids other than BHB and AcAc and is rapidly exhaled through the lungs (*p* < 0.001, Figure 3D). As a result of analysis at each time point, a significant positive correlation was also observed between TKB and breath acetone from 0.5 h to 6 h (Supplementary Figure S2).

### 3.6. Vital signs, GSRS scores, and blood glucose

No adverse events were observed from administration of ketogenic formula, and there were no significant changes in vital signs or GSRS scores (Supplementary Tables S1, S2). No significant changes in blood glucose were observed in any group after ingestion of ketogenic formula (Supplementary Figure S3).

## 4. Discussion

Ketogenic formula has long been used in the ketogenic diet for children with refractory epilepsy or congenital metabolic disorders (15). On the other hand, it was not clear how much formula was needed to increase the blood concentration of ketone bodies in adults. In this study, we found that ketogenic formula increased total serum ketone bodies, β-hydroxybutyrate, and medium-chain fatty acids (octanoic acid and decanoic acid) in a dose-dependent manner in healthy adults. Ketogenic formula also gradually increased breath acetone in all groups.

From previous studies, it is considered that these results are largely due to the contribution of MCTs contained in ketogenic formula (16–19). MCTs are rapidly digested and absorbed after ingestion and are converted to ketone bodies in the liver (12). It has been shown that the blood concentration of ketone bodies increases, depending on the intake of MCTs (20), and is elevated at low doses of MCT (17). Moreover, blood ketone bodies were shown to be increased after MCT ingestion compared with after LCT ingestion (18, 19). In another study, an MCT-supplemented ketogenic diet increased blood β-hydroxybutyrate and led to nutritional ketosis compared with an LCT-based ketogenic diet (16). Competitive interactions of MCT and LCT were also reported (21). The presence of LCT does not affect the ketogenic effect of MCT, and adding LCT to MCT increases blood ketone body levels compared with MCT alone. Among MCTs, octanoic acid has the highest ketogenic effect compared with decanoic and dodecanoic acid, and decanoic acid has a higher ketogenic effect than dodecanoic acid (11). In the present study, ketogenic formula increased blood octanoic and decanoic acids as well as ketone bodies. Moreover, a positive correlation was observed between serum TKB and octanoic and decanoic acid concentrations. Therefore, the keto-inducing effect was considered to depend on the blood concentrations of octanoic and decanoic acids.

To our knowledge, this study is the first to investigate the changes in blood levels of medium-chain fatty acids and their metabolites, ketone bodies, and breath acetone levels after ingestion of MCT, whereas previous studies investigated only changes in blood ketone bodies and medium-chain fatty acids (11, 22, 23). In this study, blood ketone bodies after ketogenic formula ingestion were paralleled by breath acetone concentration. In addition, a strong correlation was observed between serum TKB and breath acetone concentration at each time point. Although some devices for measuring breath acetone are highly variable, the device used in this study has been reported to show a high correlation with the measurement using gas chromatography (24). We further confirmed that the intra-measurement variability of the device used in this study was 4.0% via a preliminary experiment (Supplementary Tables S3). Monitoring breath acetone levels is a less invasive method than measuring ketone bodies in the blood. Therefore, it seems beneficial to predict blood ketone body concentrations from breath acetone.

In this study, no adverse events were observed with the administration of ketogenic formula in healthy adults. Our findings are consistent with those of previous studies. No severe side effects were observed after a single dose of ketogenic formula in a previous study (5). On the other hand, it has been reported that MCT induces abdominal symptoms, such as diarrhea (22). It has also been reported that emulsification of MCT causes mild symptoms (22). MCT in the ketogenic formula is emulsified with LCT and milk protein during the manufacturing process. This may have reduced abdominal symptoms even with intake of ketogenic formula in this study. Hypoglycemia, which has been consistently observed in ketogenic interventions (25), was not observed in this study. The first possible reason is that hypoglycemia was suppressed by the effect of carbohydrates contained in the ketogenic formula. Another possible reason is that the amino acid alanine derived from protein in the ketogenic formula moderated the drop in blood glucose associated with ketosis (25).

The mechanism of the ketogenic diet has been thought to be due to the combined effects of ketone bodies and glucose restriction (26). It has been thought that the ketogenic diet induces metabolic changes *in vivo* by switching the energy source from carbohydrates to fatty acids and ketones bodies, resulting in therapeutic effects (26). Recently, cellular signaling functions of β-hydroxybutyrate have been reported (26, 27), and the administration of β-hydroxybutyrate as well as the ketogenic diet has been reported to prevent or improve several diseases such as neurological disorders, cardiovascular disease, diabetic kidney disease, and cancer (7, 28–30). Ingestion of exogenous ketone salts or esters is another method for increasing blood ketone bodies. It has reported that the ingestion of approximately 14 g of exogenous ketone salts could elevate blood ketone body levels to >1 mM, which corresponds to the ingestion of 30 g of MCT (31). Also, exogenous ketone salt has not been reported to cause noticeable adverse effects such as abdominal symptoms which were noted as side effects in MCT (31). On the contrary, when exogenous ketone salts are ingested, blood ketone bodies rise rapidly, but converge in approximately 2 h (31). In the present study, the ketogenic formula was able to maintain blood ketone body levels for 6 h throughout the study period without any noticeable side effects. From the findings of this study, it is considered desirable for adults to take ≥50 g of the ketogenic formula to maintain blood ketone body concentration for a prolonged duration.

One of the limitations of this study is that the number of subjects was small. With a small number of subjects, individual differences might affect the results more than differences in the test foods. Therefore, it is possible that some indicators did not detect significant differences due to the small number of subjects. Another limitation is that this study was performed in healthy participants instead of participants with cancer, which is the population receiving the ketogenic diet. Clinical and *in vivo* studies have shown that the ketogenic diet in combination with standard therapy has the potential to enhance the antitumor effects of classic cancer therapy and increase patients’ quality of life (10, 32). The ketogenic formula may contribute to the treatment or amelioration of these conditions. In addition, the participants were limited to men in this study. The reasons for this were to adjust the body sizes of the participants and to eliminate the influence of the menstrual cycle. It has been reported that the menstrual cycle affects the energy metabolism (33), and it is possible that the menstrual cycle affects the ketone body production. Maher et al. investigated the energy metabolism with women participants to consider the estrous cycle (34). To accumulate further evidence, it is essential to acquire knowledge about the utilization of the ketogenic formula and to establish a ketogenic diet regimen that can be safely implemented for various individuals, such as women and patients with cancer and other diseases. In this study, the participants were administered only the ketogenic formula in the fasting state. The ketogenesis of MCT is suppressed by consuming carbohydrate with MCT (35). Although we have previously demonstrated that the ketogenic formula increased blood ketone bodies under conditions of a ketogenic diet (36), further studies are required to investigate how much the ketogenesis of MCT decreases depending on the amount of carbohydrates ingested at the same time.

In conclusion, the ketogenic formula was increased serum ketone bodies in a dose-dependent manner. Breath acetone was also increased in correlation with blood ketone bodies, which indicates the benefit of monitoring breath acetone levels in order to predict blood ketone body concentrations during ketogenic diet therapy. The results of this study provide new insight to establish the ketogenic diet regimen in adults.

## Data availability statement

The raw data supporting the conclusions of this article will be made available by the authors, without undue reservation.

## Ethics statement

The studies involving humans were approved by the ethical review committees at each facility of Osaka University, Osaka City University, medical institution for clinical trial implementation, and Meiji Co., Ltd. The studies were conducted in accordance with the local legislation and institutional requirements. The participants provided their written informed consent to participate in this study.

## Author contributions

KN, KH, NN, HF, YO, and KA contributed to the conception and design of the study. KH, KN, MT, MN, HS, MM, ST, and HF performed the study and organized the database. AM performed the statistical analysis. KN wrote the first draft of the manuscript. KH and RE wrote sections of the manuscript. All authors contributed to manuscript revision, read, and approved the submitted version.

## Footnotes

^1^ https://www.medience.co.jp
