# Supplementary material for: Ketogenic effects of medium chain triglycerides containing formula and its correlation to breath acetone in healthy volunteers: a randomized, double-blinded, placebo-controlled, single dose-response study
Source: Front Nutr. 2023 Sep 27;10:1224740. doi: 10.3389/fnut.2023.1224740 (PMC10566634; doi:10.3389/fnut.2023.1224740)
Supplement: Supplementary file 1 [file Data_Sheet_1.docx]

Supplementary Material

Ketogenic effects of medium chain triglycerides containing formula and its correlation to breath acetone in healthy volunteers: a randomized, double-blinded, placebo-controlled, single dose-response study

Kentaro Nakamura, Keisuke Hagihara*, Naoko Nagai, Ryuichiro Egashira, Mariko Takeuchi, Mai Nakano, Hitomi Saito, Misaki Moriguchi, Satoko Tonari, Hisako Fujii, Akimitsu Miyake, Yusuke Omae and Kinya Ashida

*** Correspondence:** Keisuke Hagihara: k.hagihara@kanpou.med.osaka-u.ac.jp

# Supplementary Figures


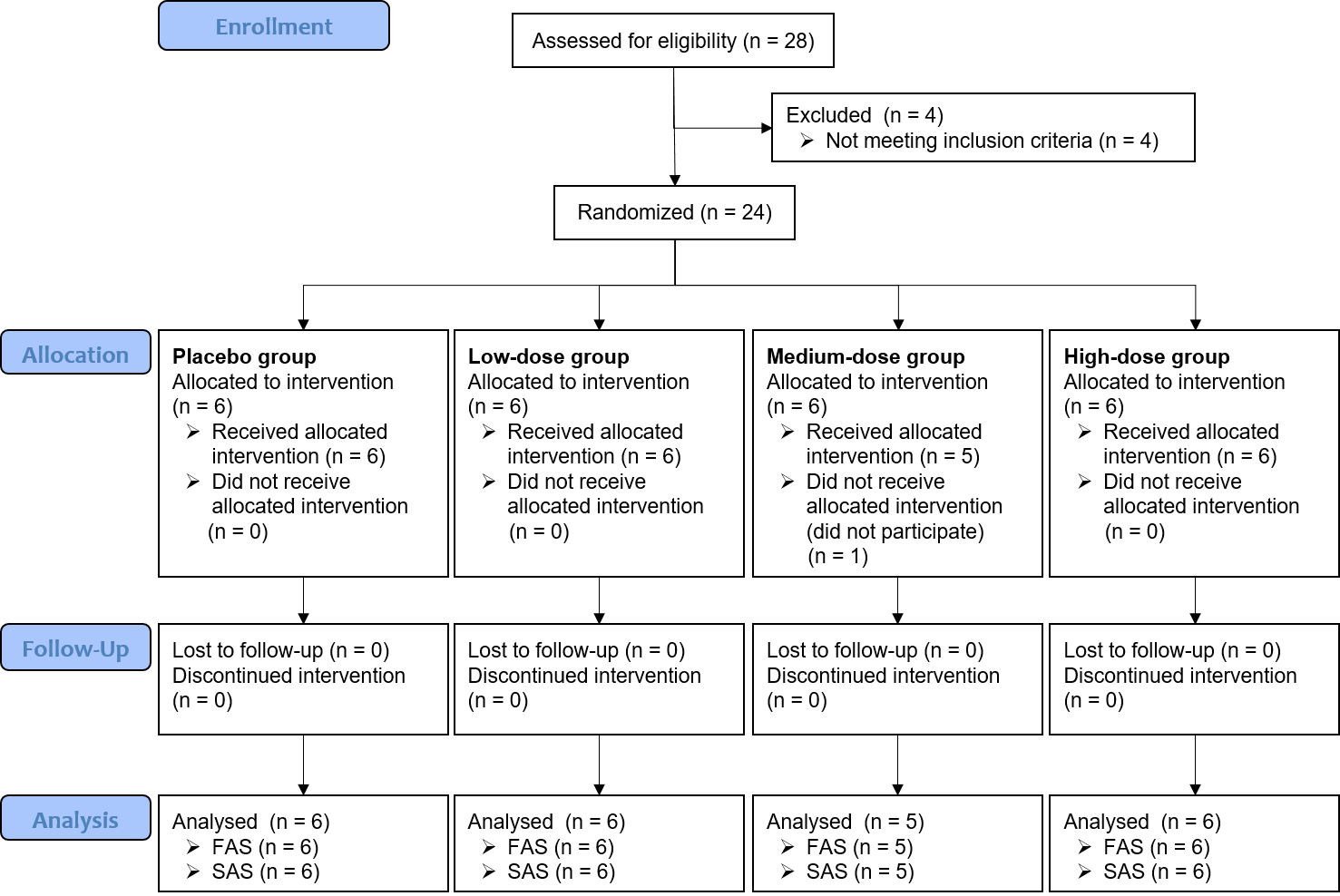


**Supplementary Figure 1.** CONSORT flow diagram


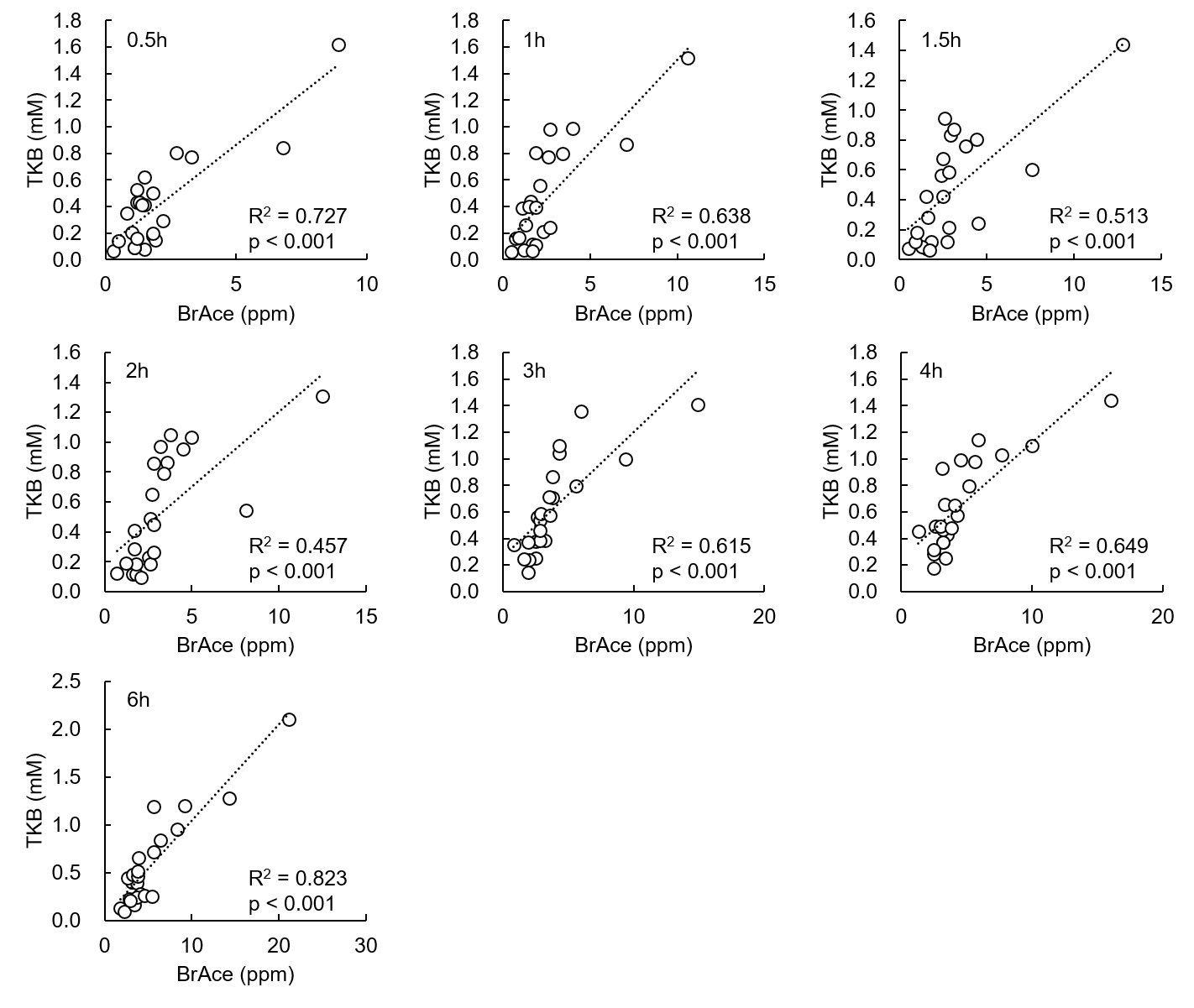


**Supplementary Figure 2.** Correlations between total ketone bodies (TKB) and breath acetone (BrAce) concentration at each time point.


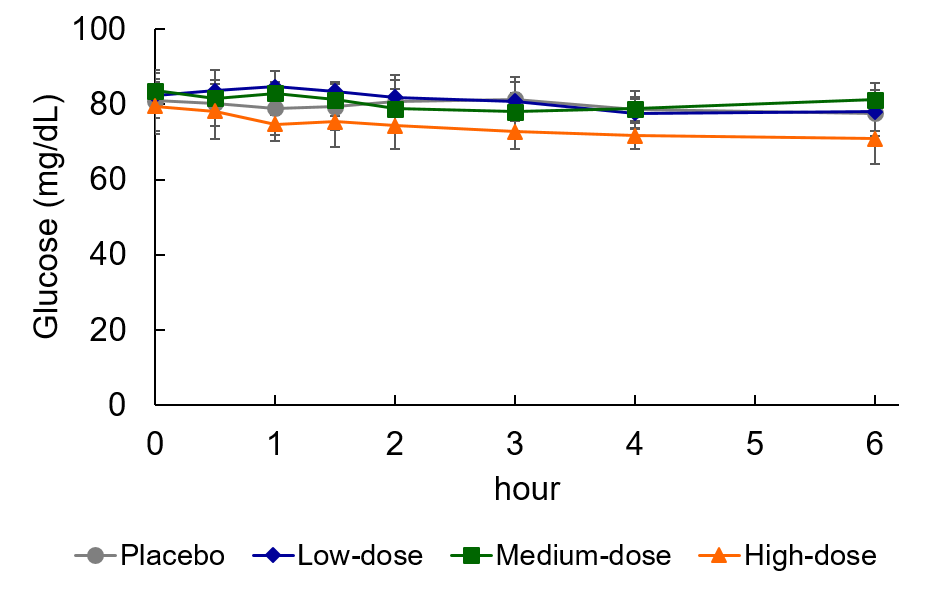


**Supplementary Figure 3.** Changes in blood glucose concentration. Values are means ± standard deviation.

# Supplementary Tables

## S1 Table. GSRS score before and after the administration of the ketogenic formula.

|  | **Placebo** | **Low-dose** | **Medium-dose** | **High-dose** |
| --- | --- | --- | --- | --- |
|  | **(N = 6)** | **(N = 6)** | **(N = 5)** | **(N =6)** |
| **Reflux** |  |  |  |  |
| Baseline | 1.08 ± 0.20 | 1.00 ± 0.00 | 1.00 ± 0.00 | 1.00 ± 0.00 |
| 6 hours | 1.08 ± 0.20 | 1.00 ± 0.00 | 1.00 ± 0.00 | 1.00 ± 0.00 |
| **Abdominal pain** |  |  |  |  |
| Baseline | 1.58 ± 0.20 | 1.75 ± 0.61 | 1.50 ± 0.00 | 1.67 ± 0.41 |
| 6 hours | 1.50 ± 0.00 | 1.58 ± 0.20 | 1.70 ± 0.45 | 1.67 ± 0.41 |
| **Indigestion** |  |  |  |  |
| Baseline | 2.67 ± 0.52 | 2.25 ± 0.42 | 2.20 ± 0.45 | 2.42 ± 0.38 |
| 6 hours | 2.75 ± 0.69 | 2.25 ± 0.42 | 2.20 ± 0.45 | 2.25 ± 0.42 |
| **Diarrhea** |  |  |  |  |
| Baseline | 1.58 ± 0.20 | 1.58 ± 0.20 | 1.60 ± 0.22 | 1.50 ± 0.00 |
| 6 hours | 1.50 ± 0.00 | 1.60 ± 0.22 | 1.60 ± 0.22 | 1.50 ± 0.00 |
| **Constipation** |  |  |  |  |
| Baseline | 1.58 ± 0.20 | 1.58 ± 0.20 | 1.60 ± 0.22 | 1.50 ± 0.00 |
| 6 hours | 1.58 ± 0.20 | 1.58 ± 0.20 | 1.60 ± 0.22 | 1.50 ± 0.00 |
| **Total score** |  |  |  |  |
| Baseline | 1.70 ± 0.15 | 1.63 ± 0.23 | 1.58 ± 0.18 | 1.62 ± 0.12 |
| 6 hours | 1.68 ± 0.17 | 1.60 ± 0.15 | 1.62 ± 0.18 | 1.58 ± 0.10 |

Values are presented as mean ± standard deviations.

## S2 Table. Vital signs before and after the administration of the ketogenic formula.

|  | **Placebo** | **Low-dose** | **Medium-dose** | **High-dose** |
| --- | --- | --- | --- | --- |
|  | **(N = 6)** | **(N = 6)** | **(N = 5)** | **(N =6)** |
| **Systolic blood pressure (mmHg)** | | | | |
| Baseline | 118.50 ± 20.73 | 117.00 ± 12.18 | 113.40 ± 9.26 | 113.50 ± 8.69 |
| 6 hours | 118.00 ± 20.71 | 118.50 ± 12.41 | 111.00 ± 8.49 | 111.33 ± 11.76 |
| **Diastolic bloodpressure (mmHg)** | | | | |
| Baseline | 67.17 ± 12.50 | 69.17 ± 5.74 | 66.80 ± 8.47 | 60.83 ± 3.76 |
| 6 hours | 64.33 ± 15.11 | 69.17 ± 7.99 | 66.20 ± 6.14 | 61.83 ± 5.91 |
| **Heart rate (bpm)** | | | | |
| Baseline | 73.50 ± 11.00 | 67.17 ± 9.99 | 64.20 ± 6.46 | 61.67 ± 3.50 |
| 6 hours | 68.67 ± 9.18 | 66.67 ± 8.62 | 60.40 ± 1.95 | 62.83 ± 6.68 |
| **Body temperature (°C)** | | | | |
| Baseline | 36.20 ± 0.41 | 36.50 ±0.28 | 36.38 ± 0.25 | 36.38 ± 0.25 |
| 6 hours | 36.43 ± 0.51 | 36.63 ± 0.18 | 36.38 ± 0.31 | 36.53 ± 0.29 |

Values are presented as mean ± standard deviations.

## S3 Table. The intra-measurement variability of the acetone monitoring device in the preliminary experiment, in which the breath acetone was measured 8h after ingesting a single dose of 20 g of MCTs.

| Number of measurement | 1 | 2 | 3 | 4 | 5 |  |  |
| --- | --- | --- | --- | --- | --- | --- | --- |
| Breath acetone (ppm) | 4.1 | 4.3 | 4.0 | 4.3 | 4.4 |  |  |
| Number of measurement | 6 | 7 | 8 | 9 | 10 |  | CV (%) |
| Breathe acetone (ppm) | 4.4 | 4.5 | 4.3 | 4.6 | 4.5 |  | 4.0 |
